# Laparoscopic Adjustable Gastric Banding and Hypoglycemia

**DOI:** 10.1155/2013/671848

**Published:** 2013-10-03

**Authors:** Sigrid Bairdain, Mark Cleary, Chueh Lien, Ashley H. Vernon, Bradley C. Linden, David B. Lautz

**Affiliations:** ^1^Department of Surgery, Boston Children's Hospital, Harvard Medical School, Boston, MA 02115, USA; ^2^Department of Surgery, Brigham & Women's Hospital, Harvard Medical School, Boston, MA 02115, USA; ^3^Pediatric Surgical Associates, Children's Hospitals and Clinics of Minnesota, Minneapolis, MN 55404, USA; ^4^Department of Surgery, Emerson Hospital-Mass General Hospital, Harvard Medical School, 133 Old Road to Nine Acre Corner, Concord, MA 01742, USA

## Abstract

Obesity is commonplace, and surgical treatment usually includes Roux-en-Y gastric bypasses (RYGBs). RYGBs have the most documented side effects including vitamin deficiencies, rebound weight gain, and symptomatic hypoglycemia; fewer series exist describing hypoglycemia following other bariatric operations. We reviewed all patients undergoing laparoscopic adjustable gastric banding (LAGB) at our institution between 2008 and 2012. Three patients were identified to have symptomatic hypoglycemia following LAGB. Mean time from surgery was 33 months (range 14–45 months), and mean weight loss was 32.7 kg (range 15.9–43.1 kg). None of the patients had preexisting diabetes. Therefore, symptomatic hypoglycemia should be investigated irrespective of bariatric operation.

## 1. Introduction

Obesity is one of the biggest health issues facing medicine. Diet and medication programs alone are often unable to maintain durable weight loss; thus, bariatric surgery has proven to provide long-term weight loss that corresponds to reduced mortality [1, 2]. The mainstay of bariatric surgery is the Roux-en-Y gastric bypass (RYGB), which has to date the most durable, documented long-term weight loss. However, long-term complications include nutrient deficiencies, hernias, excess skin, neuropathic changes, and hyperinsulinism [3]. One of the most striking side effects of this particular surgery is postgastric bypass hypoglycemia. Theories exist regarding the underlying etiology for this postgastric bypass hypoglycemia including both pancreatic-mediated (beta-cell) and nonpancreatic mediated mechanisms.

Briefly speaking, the pancreatic mediated (beta-cell) mechanisms include the following mechanisms (1) enhanced insulin sensitivity, (2) increased density of insulin islets, and (3) enhanced hormonal secretion in the bypassed limb [4]. On the other hand, nonpancreatic mechanisms have included the following mechanisms: (1) decreased levels of the appetite-stimulating and insulin counter-regulatory gastrointestinal hormone, ghrelin, and (2) alterations in other counter regulatory hormones [5]. More recently, Rabiee et al. [4] have suggested that it is the persistent exaggerated hypersecretion of GLP-1, which has previously shown to be insulinotropic, insulinomimetic, and glucagonostatic. The overexpression of the islet cell transcription factor, PDX-1, caused by prolonged hypersecretion of GLP-1 causes the hypoglycemia [4]. The most likely explanation is that it is not just one of the aforementioned mechanisms solely but a combination of beta-cell proliferation, an alteration of the hormonal milieu, and associated anatomical mechanisms [5].

## 2. Case Presentation

As a whole, there are not many recently reported cases explaining hypoglycemia following other bariatric surgical treatments, specifically laparoscopic adjustable gastric banding (LAGB). Therefore, under our own institution's review board approved protocol, we reviewed all patients undergoing LAGB between 2008 and 2012 at our institution. These patients were identified retrospectively from a prospective, longitudinal bariatric outcomes database. All patients had met the criteria for bariatric surgery established by the National Institutes of Health Consensus Development Panel, having a body mass index (BMI) of ≥40 kg/m^2^ or ≥35 kg/m^2^ with obesity-related comorbidities.

All of the patients within this program and database had at least one previously failed 6-month attempt to maintain weight loss through nonsurgical means. Nutritional and psychiatric approvals were also required; patients deemed unfit for weight loss surgery from nutritional and psychological standpoints did not receive surgery. For all LAGB cases, the Lap-Band system (Allergan, Santa Barbara, CA) was placed by experienced surgeons using the pars flaccida technique [6]. Weight loss was expressed as percent excess body weight loss [7], while overall glucose measurements were taken at the discretion of the provider.

**Figure 1:**
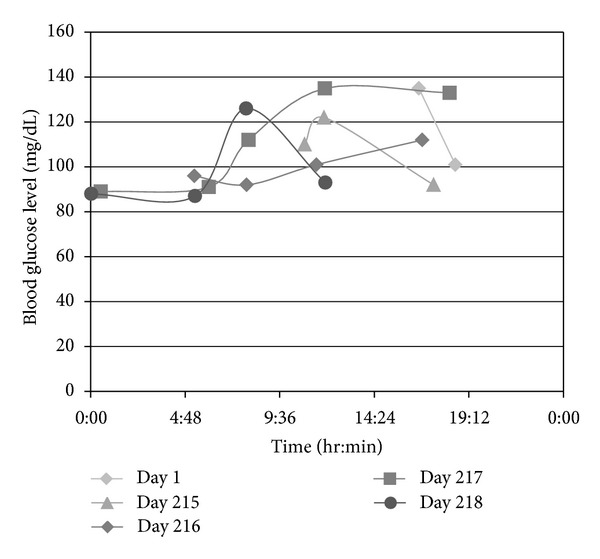
Trend in nonfasting blood glucose level of a single patient with LAGB.

Three patients were identified. Demographics and clinical characteristics of the three patients are described in Table 1. As a whole, none of the patients were on prescribed medications that may have had insulinomimetic mechanisms. As a whole, all there had a positive Whipple test. A Whipple test consists of the following entities: (1) symptoms of hypoglycemia, (2) low plasma glucose (<55 mg/dL) at the time of symptoms, (3) relief of symptoms with the correction of low glucose, as well as concomitant measurement of serum insulin (>3 *μ*U/mL), C-peptide (0.6 ng/mL), and (4) a negative sulfonylurea screen [8]. Mean age at time of surgery was 52 years (range 47–57 years). The mean time from surgery to the development of symptoms was 33 months (range 14–45 months). Mean weight loss at time of symptoms was 32.7 kg (range 21–43.2 kg). None of the patients had a preexisting diagnosis of type II diabetes mellitus (DM), but one patient did record his own blood glucose levels for trends (Figure 1).

The individual characteristics of each patient are described as follows. Patient 1 was a female. She was 57 at the time of her operation, and her preoperative profile included a body mass index (BMI) of 47 kg/m^2^. Preoperative medications included citalopram and zolpidem. The patient underwent a LAGB operation with concomitant hiatal hernia repair. Her perioperative course was benign. Symptoms appeared 45 months following the surgery. Her weight loss was 38.2 kg (84 lbs) when symptoms appeared and 0 mL was present in band reservoir.

Patient 2 was also a female. She was 47 at the time of her operation. Her preoperative profile included a BMI of 40.2 kg/m^2^. Preoperative medications included Seroquel, Wellbutrin, and Saphris. This patient also underwent a LAGB, and her perioperative course was also benign. Symptoms appeared 41 months following surgery. Her weight loss was approximately 43.2 kg (95 lbs) when symptoms appeared and 5.1 mL was present in band reservoir.

Patient 3 was a male, and this patient was 52 at the time of his operation. Preoperative BMI was 40.9 kg/m^2^. Preoperative medications included Zocor, Avalide, Tricor, and Aspirin (ASA). The patient underwent LAGB. He was admitted postoperatively for a partial small bowel obstruction (SBO); this episode resolved with nonoperative management. His symptoms appeared 14 months following the surgery. The patient was seen in the emergency room for blood glucose of 42 mg/dL which resolved with the administration of dextrose-containing solutions. At the time, his weight loss was approximately 21 kg (47.5 lbs) and 8.2 mL was present in band reservoir.

## 3. Discussion

Herein, we investigate and review the literature regarding symptomatic hypoglycemia following bariatric surgery, specifically as it relates to LAGB, as well as to bariatric surgery as a whole. This report has identified 3 patients who had symptomatic hypoglycemia following LAGB. Our patients' symptoms developed, on average, 33 months following the surgery, with the earliest symptoms being documented within the first 14 months. In this particular patient, his symptoms were not associated with his maximum net weight loss. Collectively, all three patients had similar preoperative body mass indexes (BMI), but, on average, the females (1) took a longer time to achieve substantial weight loss and (2) the development of their symptoms, from the time of the surgery, was longer.

The current literature estimates the overall incidence of reported postprandial hypoglycemia following RYGB to be approximately 1% [9]. Alterations in the volume placed within the band reservoir seemed to have no influence on the development of these symptoms, nor their frequency, in these three patients, and subsequent reports have corroborated these findings. None of the patients had preexisting diabetes mellitus (DM). This finding is congruent with previous reports from both Goldfine et al. [3] and Marsk et al. [9] who commented that there is no observable association between preoperative diagnosis of DM and the development of these symptoms. In fact, the overall proportion of persons with diabetes mellitus who undergo bariatric surgery and develop this complication is surprisingly low.

Overall, there are mixed reviews of hypoglycemic events following gastric banding procedures. We acknowledge that gastric banding does not alter absorption and is purely restrictive; yet, it has produced similar symptoms to other bariatric procedures which are both malabsorptive and restrictive. In juxtaposition to our study of three patients, Marsk et al. [9] reported that there were no appreciable differences in hypoglycemic episodes for their cohort of banding patients versus a reference cohort population. Scavini et al. [10] reviewed their 222 cases of gastric bands and found a 3%-4% (*n* = 8 patients) rate of asymptomatic hyperinsulinemic hypoglycemia; they attributed this to the reduction of body mass index (BMI) and the inherent improvement in insulin sensitivity. In review of our cohort, our patients' episodes occurred independently of their maximum net BMI reduction, and thus, may not be entirely due to an improvement in insulin sensitivity.

Only one patient in our study had a concomitant hiatal hernia repair which may have portended more manipulation of the vagus nerve, thus, inadvertent regulation of gastric hormones. Historically, procedures associated with greater manipulation of the vagus nerve have included esophagectomies, gastrectomies, RYGB, and Billroth configurations; they all reroute the foregut anatomy [11]. Interestingly enough, upon review of other reported LAGB studies, these studies have investigated long-term weight regulating effects of vagotomy when performed in conjunction with gastric banding, however, not specifically hypoglycemia [12–15].

The symptoms of hypoglycemia may be debilitating and often invoke other treatment measures; often even the treatments do not fully reverse the symptomatology. Patients are often subjected to invasive testing to determine whether there is a component of segmental hyperinsulinemic response to ingested meals; intra-arterial calcium-stimulated selective hepatic venous sampling allows the practitioner to determine whether there is a component B-cell hyperplasia with associated high calcium-stimulated insulin gradient [16].

Hypoglycemic episodes follow meals and are often resistant to changes in character of meals. All the patients reported symptoms consistent with Whipple's triad, and one patient's hypoglycemia required a subsequent admission to the hospital. A positive Whipple's triad is often inherent in cases: (1) symptoms of hypoglycemia following a meal, (2) documentation of hypoglycemia, and (3) symptoms relieved by the administration of glucose [8, 11]. For example, despite “normal glucose” values (see Figure 1), our third patient met criteria for Whipple's triad on multiple occasions. The episodes should not be confused with “reactive hypoglycemia;” these symptoms can be ameliorated with changes in diet volume and carbohydrate percentage [17, 18].

Furthermore, the timing of these symptoms does not correlate with weight loss or overall associated nutrition changes. As mentioned previously, our patients' symptoms developed, on average, 33 months following the surgical intervention, and this is congruent with other studies. Known cases of hyperinsulinemic neuroglycopenia have often developed very late, ranging from 1 to 9 years following RYGB (typically 2–4 years); at this time, cell adaptation to the postoperative milieu, as well as some renewed insulin resistance from incipient weight regain, should have reduced the likelihood of excessive insulin secretion [19].

There are a host of available treatments that are noninvasive and invasive depending on the severity and chronicity of symptoms. Available treatments include (1) very low-carbohydrate diets; (2) diazoxide, acarbose, and octreotide; and (3) subtotal pancreatectomy [16]. According to McLaughlin et al. [16], they placed a gastrostomy into the remnant stomach to alter delivery of nutrients, while not compounding the hyperinsulin versus dysregulated B-cell's response. It appears that the treatments may be suboptimal and none were applied to LAGB as it does not alter the anatomical configuration of the bowel present, nor should it alter the delivery of postprandial, regulatory hormones. As it pertains to nutritional changes and vitamin supplementation, supplements for iron, calcium, Vitamin B12, folate, and selenium should still be given to LAGB patients given the overall reduced caloric intake [20].

## 4. Conclusion

We continue to recommend that symptomatic hypoglycemia be investigated irrespective of bariatric surgery performed. Further studies are needed to ascertain the etiology of hypoglycemia in LAGB patients on a larger scale.

## Figures and Tables

**Table 1:** Case patients' characteristics.

Variables	Patient 1	Patient 2	Patient 3
Age (years)	57	47	52
Gender (male, female)	F	F	M
Preoperative BMI (kg/m^2^)	47	40.2	40.9
Medication-related complications	N	N	N
History of type II diabetes mellitus	N	N	N
LAGB placement	Y	Y	Y
Concomitant hiatal hernia repair	Y	N	N
Presence of perioperative complications	N	N	N
Time to development of symptoms (mo.)	45	41	14
Presence of Whipple's triad	Y	Y	Y
Weight loss at time of symptoms (kg)	−38.2	−43.2	−21
Volume of band reservoir (mL)	0	5.1	8.2

BMI: body mass index; LAGB: laparoscopic adjustablegastric banding; mo.: months; kg: kilograms; mL: milliliters.
